# Keeping the promise: a critique of the current state of microdosing research

**DOI:** 10.3389/fpsyt.2024.1217102

**Published:** 2024-02-05

**Authors:** Rotem Petranker, Thomas Anderson, Emily C. Fewster, Youval Aberman, Marik Hazan, Michael Gaffrey, Paul Seli

**Affiliations:** ^1^Department of Psychology, Neuroscience and Behaviour, Hamilton, ON, Canada; ^2^Department of Psychology, University of Toronto, Toronto, ON, Canada; ^3^Independent Researcher, New York, NY, United States; ^4^Department of Psychology and Neuroscience, Duke University, Durham, NC, United States

**Keywords:** psychedelics, microdosing, review, psilocybin, LSD

## Abstract

**Introduction:**

The practice of taking small, sub-hallucinogenic doses of psychedelics, known as microdosing, has exploded in popularity over the last decade. Users claim benefits ranging from improved mood and enhanced creativity to an increased sense of meaning and connectedness in life. While research on microdosing is still lagging behind the shift in public opinion, several papers have been published in the last five years which attempted to assess the effects of microdosing.

**Methods:**

This review paper aimed to critically analyze the research practices used in the recent wave of microdosing research: We reviewed 15 papers published before the closing date of this review in March 2022.

**Results:**

Our review concludes that it is premature to draw any conclusions about the efficacy or safety of microdosing since the research quality cannot be considered confirmatory.

**Discussion:**

We propose some potential causes for the current state of the literature and some suggestions for how these causes may be ameliorated.

## Introduction

In the last few decades, psychedelics have returned to the spotlight of public attention in what has been referred to as the *Psychedelic Renaissance* (1). Evidence suggests that hallucinogenic doses of psychedelics may have positive effects on mood, creativity, and a host of other indices of human well-being and performance (see Lowe et al. (2) for a review). Microdosing—the practice of taking small, sub-hallucinogenic doses of psychedelics—has attracted much attention from researchers in recent years (3), but the research has yet to catch up with the enthusiasm. The practice of microdosing is particularly interesting for two main reasons. First, if the positive impact of psychedelics can be attained without the difficult psychological experiences that are known to sometimes accompany larger doses (4), patients may prefer to microdose instead. Second, if microdosing has discernible psychological effects, this finding would challenge current theories about the necessity of mystical experiences in facilitating the putative effects of psychedelics (5). Microdosing seems promising for improving mental health and various aspects of well-being, but the amount and quality of research on its benefit are arguably lacking (6).

Over the last decade, roughly 20 experimental or quasi-experimental papers on microdosing have been published. These studies have used various methods ranging from ethnographic analysis to double-blind placebo-controlled studies. Despite efforts to establish convergent evidence derived from numerous different analytic and methodological approaches, conflicting findings have been reported. Overall, there is scant research on microdosing, but the research tends to suggest that microdosing is effective and beneficial (7). However, puzzlingly, microdosing appears to show contradictory effects, for instance, in that it has been found to both increase and decrease mood, anxiety, headaches, and attention [for examples, compare (8, 9)]. One possible explanation for these conflicting findings is the general lack of consideration of the mental set and environmental setting when microdosing, both of which may cause a high degree of variability in outcomes [see (10)]. For example, microdosing under stressful conditions may induce anxiety, while microdosing under stress-free conditions may alleviate anxiety. While plausible and useful, this theory still leaves many of the different effects unexplained. In addition, since microdosing is a relatively new area of research, there is a need for new research and studies that are mainly focused on detecting any effect at all. This artifact of the publication and academic systems in which scientists are incentivized to publish interesting results rather than rigorously examine the impact of the intervention they study leads to a gap in our understanding of microdosing. While it is possible that this is not the case in psychedelics, similar trends have happened before, for example, in the study of mindfulness (11).

The gaps in our understanding of microdosing due to conflicting findings have at least three possible negative outcomes. First, they can create an unsound basis for future research, which could lead the field to another replication crisis, only this time one that (a) is specifically pertinent to psychedelics research, and (b) may have a negative impact on the legality of these substances (3). Second, These gaps have invited various interested parties including self-help gurus who confidently assert that their way of microdosing is the “right” way without scientific support (12) or grounding in historical grounding (13). Third, in the absence of valid and reliable research on microdosing, users may embrace ineffective, costly, and potentially dangerous microdosing practices that do not consider individual differences between users. There is much unknown about microdosing and its effects, and at least some of the gaps in our knowledge have arguably been born from our attempts to develop the best practices for this nascent field.

One way to improve this general state of affairs is to produce sound, reliable, open scientific evidence on both the positive and negative outcomes of microdosing. Some guidelines—including those outlined by the principles of Open Science—include pre-registration, the open sharing of data, and constraints on generalization, all of which are always important, but particularly so in the context of the nascent field of psychedelics (3). Another way to improve the literature is to critically review its current state, focusing on publications pertaining to microdosing psychedelics and teasing out trends, strengths, and weaknesses. Others have done an excellent work of collating and explaining the findings in the current wave of research (7) and have looked at larger historical perspectives and critiqued the current state of the research (6).

Building on this important work, here, we aim to assess the fidelity, reliability, and replicability of the current findings in microdosing research. We argue that transparency is necessary to produce high-quality data, replicable analyses, and the accurate interpretation of results. Relatedly, we argue that since, in recent years, transparency has not been strongly encouraged in psychedelics research, the quality of the scientific outputs in this domain could be improved by drawing researchers’ attention to the importance of transparency in scientific investigations. In addition, we highlight some of the weaknesses in published studies on microdosing in terms of their design, analysis, and inferential logic, and we draw on these to help to explain some of the conflicting findings in the literature. The key themes we discuss include (a) a general lack of open-science practices (including a lack of pre-registration, open materials, and open data), (b) biased samples, (c) questionable methods and analyses, (d) the drawing of inappropriate inferences from the data, and (e) irreproducible analyses and findings. Despite the issues we raise in this paper, we believe that the overall picture suggests that microdosing is a promising practice, and hope that future research will answer some of the questions left open by the papers reviewed here.

## Methods

In early 2022, we contacted, via email, corresponding authors of experimental and quasi-experimental microdosing papers and requested their data to reanalyze and reproduce their findings. We included papers reviewed in Ona and Bouso (7); moreover, since the field is rapidly changing, we contacted the author of another paper (14), published between the publication of Ona and Bouso (7) and the writing of this manuscript.” We did not, however, contact authors whose datasets are freely available online (we instead simply downloaded their data for re-analysis). In deciding which work to include in our review, we set a cut-off date for paper publication for March 1st, 2022, and we requested that data be shared with us by May 1st, 2022. The papers whose datasets we acquired, whose results we attempted to replicate, and the availability of the data are reported below (see Table 1).

**Table 1:** Papers included in this review, data availability, and journal guidelines for data availability.

Study	Journal	Journal guidelines for data availability	Response	Notes
Data available online				
Van Elk et al. (15)	Psychopharmacology	Encourages	N/A	
Szigeti et al. (16)	eLife	Expects	N/A	
de Wit et al. (14)	Addiction Biology	Expects	N/A	
Marschall et al. (17)	Journal of Psychopharmacology	Expects	N/A	
Data shared by authors				
Bershad et al. (18)	Biological Psychiatry	Encourages	Shared data	Shared partial dataset
Bershad et al. (19)	Biological Psychiatry: Cognitive Neuroscience and Neuroimaging	N/A	Shared data	Shared partial dataset
Data not shared by authors				
Prochazkova et al. (20)	Psychopharmacology	Encourages	No response	
Yanakieva et al. (21)	Psychopharmacology	Encourages	No access to data	Data with pharma company, no response
Madsen et al. (22)	Neuropsychopharmacology	Expects	No access to data	Data with 2nd author, no response
Family et al. (23)	Psychopharmacology	Encourages	No access to data	Corr. Author not part of research team
Ramaekers et al. (24)	Journal of Psychopharmacology	Expects	Refusal	
Hutten et al. (25)	ACS Pharmacology & Translational Science	Expects	Refusal	
Hutten et al. (26)	European Neuropsychopharmacology	N/A	Refusal	
Holze et al. (27)	Clinical Pharmacology & Therapeutics	Expects	Refusal	

N/A means not applicable.

Using the datasets that were shared with us, and those that were openly available, our approach was to attempt to replicate only key analyses from the original papers, focusing our attention on the specific methodological, statistical, and logical considerations in the papers we examined. To minimize bias in the analysis process, one of the authors (YA)^1^, communicated with corresponding authors and performed all analyses. YA was asked to repeat analyses that he considered to be crucial in supporting the conclusions of the papers reviewed here; any other impressions that YA may have had about the papers were withheld. Below, we detail our findings according to themes that emerged from the papers we reviewed.

## Results

Seven themes emerged during our review of the experimental and quasi-experimental studies based on our understanding of the scientific method and the guidelines suggested by Munafò (28). These themes were (a) open science (pre-registration and data sharing/openness of materials), (b) design quality, (c) sampling strategies, (d) dose accuracy, (e) appropriateness of measures, (f) appropriateness of analyses, and (g) appropriateness of the inferences from results of data analyses.

### Lack of open science

In this section, we aim to succinctly explain what the practice and theory of Open Science is in general and briefly exlplain the benefits of following this framework. We then apply it to the context of microdosing research and assess whether the papers reviewed here satisfy the core tenets of Open Science, with a particular focus on data sharing. We end with suggestions for future research.

Open Science is the practice of making scientific research—including study plans, methods, analyses, raw data, and publications—transparent and publicly available (29). There are several advantages to using Open Science. First, through transparency, other scientists can critically assess the research practices of one’s work such that peer review becomes a more rigorous and ongoing process. Second, Open Science allows consistent reproduction of study methods, which in turn facilitates replication attempts and exploratory analyses of existing datasets, thereby making research more useful to the scientific community. Third, publicly available research tends to be more impactful (30), perhaps because stakeholders outside of academia have direct access to primary resources; consequently, policymakers, educators, clinicians, and the general public can directly be informed by research. Fourth, Open Science encourages wider collaborations, which are quite helpful in tackling larger research questions. Finally, given that the majority of scientific research is publicly funded, the public arguably deserves access to the research and its results (31) (although in the case of privately-funded research, there is an argument to be made that the data belongs to those who funded it, if such conditions are agreed upon). All of these advantages are particularly relevant for the nascent field of psychedelic research (3).

Among the 14 papers surveyed in this review, only two were pre-registered and both papers reported on data from the same study (15, 17), and only Marschall et al. (17) clearly delineated the constraints on the generality of their findings, which is an integral part of current best practices in psychedelic science; Petranker et al. (3). Moreover, despite journal guidelines either “encouraging” or “expecting” the sharing of data, data from only four out of 14 papers were available online (14–17). The responses (or lack thereof) to our requests for the data from the remaining ten papers were inadequate for replication (see Table 1 for data availability by paper). Of the 14 papers surveyed here, two incomplete datasets were provided, but were without coding keys, and the researchers unfortunately did not respond to our requests for the complete datasets and coding keys. One author reported that a pharmacology company owns their data; they asked for additional time, then stopped responding to email. One author was away and their alternate contact did not respond to our requests. One author asked to confer with their coauthor, then did not respond to our follow-up emails. The remaining five papers were authored by the same research group; however, the authors refused, via email response, to share data without providing a reason for refusal. See Table 1 for a summary of these results. The reproduction of key analyses in the four publicly available datasets (14–17) was completed successfully, replicating the key findings reported in these papers. Our reproduction of Bershad et al. (18) and Bershad et al. (19) was limited by missing essential variables in the provided datasets. Bershad et al. (18) only contained two of the four primary self-report outcomes, which were the primary outcome variables in the paper, but none of the behavioural/cognitive measures. Bershad et al. (19) did not contain the primary dependent variable (imaging data), but as our replication focused on self-reported data, this was no hindrance to our reproduction attempt. Overall, the reproduction for all these papers did not reveal any significant disparities between the reported analyses and our outcomes.

In considering the general lack of open-science practices engaged in the papers examined here, we suggest that, moving forward, researchers of microdosing should seriously consider engaging in such practices (i.e., pre-registration, sharing of materials and data, and reporting constraints on the generality of one’s findings). Moving forward, we suggest that the practices of Marschall et al. (17) —which was pre-registered, allowed open-access to data, and reported constraints on generality— will serve as a good guideline for researchers to use when creating their study plans, analyzing their data, and drafting manuscripts.

### Design quality

Here, we discuss some basic concepts of research design in general and then focus on blinding and set and setting which are of particular import for microdosing research. We examine the design quality of experimental and quasi-experimental studies separately. Finally, we end with additional recommendations for future research based on the current state of the literature.

Blinding (the process of making the experimental intervention unknown to participants in a study) is important when performing experiments on new interventions whose impact is not yet entirely known. By blinding participants and researchers to experimental and control conditions, it is possible to reduce a variety of biases, including observer bias, confirmation bias, and a disproportionately large placebo effect (32). Blinding is of particular relevance in the study of psychedelics, in which *set and setting* are canonically major confounding factors (33). While blinding in high-dose trials can be quite difficult (because of the obvious perceptual effects of psychedelics), blinding in microdosing studies should theoretically pose less of a concern given that the influence of the substance is intended to be subtle and sub-hallucinogenic. As breaking blind (that is, participants becoming aware of whether they area in the experimental or control condition) may cause unreliable response patterns and incorrectly estimated effect sizes, unblinded designs can be useful for producing preliminary or pilot data, but are inappropriate for confirmatory analyses (34). In this section, we discuss blinding and breaking blind across the 14 studies we reviewed, dividing these studies into those that were *experimental* and those that were *quasi-experimental.* It is important to note that it is possible that in some cases blind was not broken, hence the lack of reporting about breaking blind. At the same time, it is also possible that breaking blind was not reported because the standard for reporting this variable has yet to be established.

The ten *experimental* papers we reviewed used double-blind, placebo-controlled designs, with either within-subject (15, 17–19, 25, 35, 36) or between-subject (14, 21, 23, 24) experimental designs. Madsen et al. (22), a pharmacokinetics and pharmacodynamics study, included eight blinded participants and is included here for completeness. Only two of these papers (15, 17, 27) reported rates of participants breaking blind.

The four *quasi-experimental* papers we reviewed used either no blinding (20) or *ad-hoc* self-blinding (15–17). Szigeti et al. (16) used a clever design that instructed participants on how to create randomized placebo and experimental packets to be used during the study. Only three of the quasi-experimental studies (15, 16, 25) reported the rates of participants breaking blind. In all three studies, blind was broken at rates higher than chance, raising concerns about papers that failed to report rates of breaking blind.

Notably few papers reported consideration of set and setting despite the importance of such factors when using psychedelics (33, 37) and their potential importance in microdosing (16, 38). Family et al. (23), Madsen et al. (22), and van Elk et al. (15) mention the mental set and physical setting in which participants were dosed—which may have an important impact on outcomes, especially in microdosing—but most of the papers reviewed here did not provide these details. As the set and setting under which microdoses are consumed may be a relevant for therapeutic outcomes, the lack of consideration of such factors may be a source of confounds in the microdosing research reviewed here (10).

While online surveys have been useful for obtaining information about difficult-to-reach populations, we recommend that future research focus on double-blinded studies in which confounding variables can be better controlled. While there are many potential confounding variables rearing their heads in research on psychedelics, two key considerations (where possible) should be (a) the mental set with which participants approach microdosing (e.g., expectancy, daily mood) and (b) the physical setting in which the microdoses are consumed. Longitudinal designs are also preferable to cross-sectional designs as they more closely mirror the practice of microdosing in real-life use (8), although material and financial constraints often prevent researchers from engaging in longitudinal studies. We therefore suggest that, after conducting a controlled study, researchers continue to engage participants in online surveys, which are inexpensive, so that they may measure whether there are any longitudinal effects attributable to microdoses after participants no longer consume the substances. Finally, participants should be asked whether they think that they are in the experimental or placebo group and what evidence they have for this observation; these rates and responses should be reported (39).

### Sampling strategies

This section discusses the various constraints involved in adequately recruiting and sampling populations for microdosing studies. We critically examine how participants were recruited, which participants were recruited, and whether the results obtained from these participants are generalizable to the general population. We first examine sample size, and then past experience and motivation. Next, we specifically focus on the population and whether it is representative of the general population. Finally, we make suggestions as to how future sampling strategies could be more effective.

All papers reviewed here had samples with the standard limitations that accompany ecologically valid studies. Szigeti et al. (16) and Marschall et al. (17) were the only papers with sample sizes larger than 30 participants, although the designs of some papers compensate for the small sample size by repeated sampling (40). However, the small sample sizes are also of note because of the low likelihood of detecting adverse events with such small samples (41). all other papers included relatively small sample sizes or populations, meaning that generalizations to the general population may not be appropriate/possible [e.g., aged 55 and older, Family et al. (23) and Yanakieva et al. (21) experienced hallucinogen users, Prochazkova et al. (20)]. The remaining papers examined here reported on unblinded studies.

Almost all studies recruited participants who were experienced with psychedelics. Most had “at least one previous experience with a psychedelic drug/hallucinogen” as an inclusion criterion. Participants in van Elk et al. (15) and Marschall et al. (17) were recruited at a “Microdosing Information Workshop,” and participants in Prochazkova et al. (20) were recruited at a “microdosing event,” both of which were organized by the Psychedelic Society of the Netherlands; sampling participants in this way cannot provide a random sample due to strong self-selection pressures guaranteeing an existing interest in psychedelic microdosing prior to the study. To clarify, we believe that participants in an unblinded study who are likely to have a bias to report inflated effects are likely to produce inflated evidence (38), and should therefore be inferred from cautiously.

In a sample of older adults, Family et al. (23)/Yanakieva et al. (21) reported “LSD experience within the past 5 years” as an exclusion criterion, but this does not mean that participants had never had experience with a psychedelic/hallucinogen. Overall, biased samples lower the quality of research and introduce confounds (42). For example, it may be that participants who are experienced with psychedelics display particular response biases and may have been more aware of psychedelic activity, allowing them to break blind more often than psychedelic-naive participants; this would accord with the high rates of breaking blind seen in studies that reported such rates.

Moving forward, we suggest that future research consider performing power analyses prior to recruitment to assess the sample size needed to detect the hypothesized effect size or the minimum effect size of interest. Some researchers may already run *a priori* power analyses, but refrain from publishing them; we recommend that all *a priori* power analyses be published alongside the analysis of data. Additionally, while some research will focus on specific demographic groups, we recommend recruiting diverse participants and including participants with no previous experience using psychedelics. Once standards have been established for whether previous experience with psychedelics is a confounding factor for microdosing trials, new experimental paradigms should be developed to accommodate these findings.

### Dose accuracy

In this section, we discuss the accuracy of doses administered in microdosing trials and the reasons of the importance of dose accuracy. We first discuss LSD research and its accuracy, and then discuss psilocybin research and its accuracy. We end with a section suggesting ways in which future research may have better dosing strategies.

A core aim of psychedelic microdosing is ingesting a dose that is sufficient to produce measurable effects, but not so high a dose that it causes hallucinogenic effects (43). With this goal in mind, dose accuracy should be of paramount importance in the study of microdosing. Accurate doses should help avoid participants breaking blind (by minimizing outcomes wherein participants are explicitly aware of phenomenological changes caused by the substance), adverse events caused by “surprise trips” (i.e., when the user intends to have a sub-perceptual dose but inadvertently experiences a perception-altering effect) inflated or deflated effect sizes, and inconsistent response patterns. The latter is most likely to occur in the study of psilocybin-containing mushrooms since the concentration of psilocybin in the same fruiting body may vary up to 400% (44). Sclerotia, which are generally used as “truffles,” show the same trend (45). Thus, two individuals consuming parts of *the same mushroom* may consume vastly different doses and subsequently have vastly different experiences. Furthermore, participants may be more likely to break blind at higher rates if doses are higher than the common definition of a microdose: Indeed, when consuming higher doses, subjective effects make blinding difficult (46). While the exact dose range for a microdose is yet unknown, the generally accepted definition is 1/10th – 1/20th of a recreational dose (8). By this definition, a microdose of LSD is approximately 10–20 micrograms and a microdose of psilocybin is approximately 1–2 milligrams. However, others have suggested a broader range of doses, between 6–20 micrograms of LSD and between 0.8-5 mg of psilocybin (6).

Except where noted, the LSD research we reviewed measured doses to the microgram and provided microdoses within the aforementioned range. Bershad et al. (18) and de Wit et al. (14) included doses of 26 micrograms, which falls slightly above the aforementioned range for microdoses. Szigeti et al. (16) used participant estimates of the doses they used at home, appropriately noting in the limitations section that the “nature, purity, and dosage” were unknown.

The psilocybin research reviewed here presents a more complex trend. For example, van Elk et al. (15) and Marschall et al. (17) used 0.7 grams of dried truffles, with samples sent to further chemical analysis. However, since the variance of psilocybin in fruiting bodies can vary up to 500% (Moss et al., under review), these samples may not accurately represent the amount of psilocybin in the mushrooms consumed. Prochazkova et al. (20) suggested rough guidelines to participants regarding the amount of ground truffles to consume, but whether or not participants followed those guidelines remains unknown; indeed, the actual self-administered dose was not reported in the paper and may not have been measured at all. Prochazkova et al. (20) also sent samples for analysis, but the same critique regarding the variability of psilocybin content levelled above is also applicable here. In contrast, Madsen et al. (22) used a specific weight-adjusted dose of psilocybin, reaching very accurate levels of psilocybin administration; their dose was three milligrams, which falls slightly above the aforementioned range for microdoses. It is important to note, however, that despite accurate dosing, predictors of psychedelic experience remain unknown, and weight-adjustment appears to be irrelevant.

Future psilocybin research could use synthetic psilocybin or a standardized, homogenized extract, ensuring participants get exactly the same amount of active substance. The question of how different individuals metabolize and experience psychedelics remains open (47), but administering consistent doses of any drug is a basic requirement for pharmacological research. In addition, more research on the predictors of dose–response and the relevant dosage for various indications should be undertaken.

### Appropriateness of measures

In this section, we discuss the specific measures used to study the effects of microdosing and their fit to the theoretical construct examined and other concerns related to the validity of the measure. We mention the adequacy of the measures used in the papers discussed in this review, starting from the most well-established measures. At the end of this section we make general suggestions about how measure selection could be more adequately done in the future.

Some of the studies reviewed here used unorthodox measures for the variables they wished to examine, whereas others were careful to use well-established measures. Marschall et al. (17) used a standard cognitive depression self-report scale (DAS), an interoception scale (MAIA), and a go/no-go task. Ramaekers et al. (24) used a widely-used task to measure pain tolerance and other inventories. Szigeti et al. (16) used largely well-validated and widely used measures, though this paper used an outdated version of the Cognitive and Affective Mindfulness Scale (CAMS) (48), which was revised in 2016 (49). Bershad et al. (18) used exclusively well-validated measures, although the International Affective Picture Task has been criticized in the last decade as having low ecological validity (50).

Hutten et al. (25) mostly used well-validated measures for their variables of interest. In their case, the exceptions were the “Cognitive Control Task” (51) and the Ego Dissolution Inventory (52). The Cognitive Control Task is not a standard attention task, but it may be applicable in this context since there is an argument to be made that the researchers were interested in examining cognitive flexibility. However, well-validated tasks exist for this purpose (51). The Ego Dissolution Inventory is primarily used for research with high doses and did not show sensitivity to various doses of alcohol or cocaine (52); it is unlikely that this measure is sensitive enough to capture subtle changes that may be elicited by low doses of psychedelics. This paper otherwise used well-validated measures, including the Psychomotor Vigilance Task (PVT), the Digist Symbol Substition Test (DSST), Profile of Mood States (POMS), and the Groninger Sleep Scale (GSS).

Family et al. (23) mostly used well-validated methods to measure their variables of interest. A notable exception is their measurement of subjective effects: this paper used subjective effect measures that were originally developed for assessing either the effects of high doses of a different substance (MDMA) (53) or high doses of LSD (54). It is possible that these scales lack the appropriate sensitivity to assess the effects of much smaller doses or of different substances for which they were not designed. In a similar vein, Yanakieva et al. (21), which may have used the same data set, examined the subjective perception of time using a task developed for large doses of LSD (55). This task asks participants to memorize the amount of time required for a circle to expand to a certain size, then press a key after the same amount of time has subsequently elapsed. This measure is likely confounded with attention, decision-making, and short memory (55), such that this task has questionable validity as a measure of time perception.

Madsen et al. (22) employed more oft-used MEQ30 and EDI, which as noted above, may not be sensitive enough to detect the effects of very small doses of psychedelics.

Some studies used even more-unconventional methods. Van Elk et al. (15), for instance, used an “awe manipulation” in which participants consumed a microdose and were then presented with several videos, reporting their feelings of awe with the goal of determining whether participants felt more awe under the effect of a microdose. This intervention was not validated and was only used in previous work by the same group (56), making it difficult to assess the results. The same study also used an unvalidated art perception task in which subjective impressions of the profoundness of the art presented were measured, as well as positive and negative emotions elicited by the art. This task could have psychometric issues so results should be interpreted cautiously (57).

Prochazkova et al. (20) reported using the Picture Concept Task (PCT) from the WISC-IV intelligence test for children as a creativity measure. The paper claims that this is a good measure of “convergent creativity,” but the WISC-IV manual describes the PCT as measuring “categorical, abstract reasoning” (58). Some of the measures used in this paper were well-validated, including the Alternate Uses Task and Raven’s Progressive Matrices.

Holze et al. (35) used an eclectic mix of measures for subjective effects. This paper used the somewhat dated, infrequently used measure of mood (the Adjective Mood Rating Scale; AMRS), the States of Consciousness Scale Questionnaire (SCS) (59) that includes 43 items regarding Mystical Effects (MEQ-43) and 30 items about Mystical Effects (MEQ-30), as well as subscales for “aesthetic experience” and negative “nadir” effects. It is of note that this scale was later revised to become the shorter and more psychometrically sound MEQ-30 (60). This paper additionally used some subjective visual analogue scales sampled before and after the drug administration. In contrast, this paper also used some measures such as the 5D-ASC, which is well-validated in psychedelics research (61).

Finally, de Wit (14) failed to explain a number of the tasks they used. Our email inquiry for further information was unfortunately declined, with the author citing a lack of resources to assist with our request. Consequently, despite the online availability of their data, we were unable to critically assess the appropriateness of the measures used in this paper.

In the future, we encourage psychedelic scientists to use conventional, well-validated measures and to collaborate with other researchers when selecting measures. Since psychedelic science is somewhat controver and stakeholders are reasonably skeptical of the objectivity of psychedelic researchers, using rigorous measures has become even more important. Using conventional, well-validated measures would also make comparing results across different studies easier, facilitating future meta-analyses (in the discussion section, we recommend additional collaborative solutions). To clarify, we are not suggesting that no new measures should be developed; instead, we suggest that developing these new measures should be done cautiously and in line with discipline-specific standards for measure creation.

### Appropriateness of analyses

This section discusses the adequacy and accuracy of the statistical methods used in the studies reviewed here. As in the previous section, this section begins from the papers which in our opinion had the strongest, most appropriate use of statistical analyses. In contrast to the previous sections, our suggestions for future practices are embedded in each paragraph, as each study had its own set of circumstances.

All papers that we reviewed used frequentist statistics with varying degrees of accuracy. While we acknowledge that experimental designs are difficult and costly to manage, we propose that some of the findings in the literature remain uninterpretable because of the statistical analyses performed. At the same time, it is clear that efforts were made to create as much knowledge and publish as many papers as possible from the available data.

Marschall et al. (17) reported both frequentist and Bayesian analyses, noting that only the former were pre-registered but that the latter add more nuance in quantifying the relative evidence. While using a mix of frequentist and Bayesian analyses is increasingly a standard best-practice in psychological research (62), this study is the only one in the literature using this best practice. The paper also adequately reports which trials were excluded (e.g., go/no-go task responses above two standard deviations from the participant’s average). Moreover, the paper appropriately clearly separates confirmatory from exploratory analyses. In the context of pre-registered research corrections for multiple comparisons are not required, and so that lack of post-hoc corrections is not an issue. The authors took care, however, to run post-hoc power analyses and correct for multiple comparisons in the exploratory analyses section, although *post hoc* analyses may not be informative (63). Additionally, this paper only reports that it was part of a larger collaboration which produced two other papers only briefly under the *Doses* section of the Results rather than more explicitly in the introduction or methods sections.

Van Elk (15) used inferential statistics on a sample of 30 participants, in a within-participant design, which is the minimum sample size canonically required for such analyses (64). It is of course challenging and expensive to run participants in a clinical trial, but the small sample size is of note because it limits the power to detect an effect. This paper followed its pre-registration, although some of the results are also presented in Marschall et al. (17), and its confirmatory and exploratory analyses are adequately, if only nominally, demarcated. Additionally, this team performed a post-hoc correction for multiple comparisons, and candidly reported when effects did not survive the corrections. This paper probes the non-significant relationship between awe and trait absorption for a mediation without bootstrapping, which suggests that the result of the mediation analysis may be inaccurate, since probing nonsignificant mediation should be performed with bootstrapping (65).

Szigeti et al. (16) tested 12 covariates in addition to their reported planned change over time from baseline to T + 5 weeks and baseline to T + 9 weeks, with additional between-group comparisons at T + 5 weeks and T + 9 weeks on a variety of measures [see Table 1 in Szigeti et al. (16)]. To control for possible expectancy effects, this study appropriately asked participants to guess whether their dose was a placebo, and took care to explore the impact of other variables on participants’ likelihood of breaking blind.

Prochazkova et al. (20) initially ran analyses on possible covariates including body weight, ingested dose, and prior experience with psychedelics. They otherwise performed simple paired *t-tests* pre- and post-intervention for most of the variables of interest, and a repeated-measures ANOVA for Alternate Uses Test scores. This paper does not report any corrections for multiple comparisons, which means that the reported effects are more likely to be false positives.

Bershad et al. (18) reported performing a repeated-measures ANOVA with dose and time as a within-subjects factors. Additionally, this paper noted that “missing cases… were deleted list-wise, which led to smaller sample sizes for some analyses.” Since this study had 20 participants altogether, the implications for the experimental power available for certain analyses are considerable, but the authors do not clearly report how many cases were deleted for each analysis. Additionally, while it is difficult to assess exactly how many analyses this paper performed, there is no mention of any post-hoc correction for multiple comparisons. It is also unclear if this sample is the same as the one in Bershad et al. (19), where additional analyses were performed. If this is indeed the same sample, it would further inflate the risk of Type I errors. Regardless, the amount of resting fMRI data used in this sample was likely too small to permit a reliable estimate of functional connectivity, and the methods reported in this paper for removing systematic and spurious effects of movement are not aligned with the field’s current best practices (66, 67).

In contrast, although Yanakieva et al. (21) and Family et al. (23) were published using the same dataset, the latter paper clearly notes that a subset of the results was already published. Yanakieva et al. (21) also report testing any of the assumptions required for linear regressions. This paper reports demographic and subjective information, as well as an exploratory analysis of the impact of microdoses on participant performance on a temporal reproduction task. The analytical approach for demographics and task completion times was a between-participant ANOVAs with chi-squared tests to further clarify group differences. In terms of task performance, this paper used linear regressions to compare different doses. The many analyses performed to probe apparent relationships in this paper were exploratory, which may explain why no post-hoc correction for multiple comparisons was performed. Family et al. (23) report the standard measurements for a Phase I trial, which we do not assess here: pharmacokinetics, pharmacodynamics, blood plasma level of LSD over time, and ECG measurements. The analysis strategy for CANTAB, 5D-ASC, and proprioception tests are of interest; however, the paper reports performing one-way ANOVA analyses followed by Bonferroni post-hoc tests on the CANTAB assessments, a 2-way repeated-measures ANOVA for the 5D-ASC, and a repeated-measures mixed model analysis for the proprioception test. It is of note that only three questions from the 5D-ASC turned out significant, suggesting that analysis was performed for each individual item, but the authors do not report post-hoc corrections for this outcome.

Similarly, Hutten et al. (25, 26) also note that the two papers report on data from the same study. We do not discuss the results from Hutten et al. (25) here since they are primarily about the pharmacodynamics and pharmacokinetics of small doses of LSD. Hutten et al. (25) note that they tested for sphericity but not other assumptions of linearity. They reported baseline-correcting some variables before entering the statistical analysis. They then analyzed the Psychomotor Vigilance Task (PVT), Digit Symbol Substitution Test (DSST), Profile of Mood States (POMS), and Visual Analogue Scales (VAS) regarding their experience using a General Linear Model ANOVA including four dose levels and 24 participants as random factors. The GSS, 5D-ASC, EDI, and CCT were assessed once for each test day and analyzed using GLM Repeated Measures ANOVA. Missing values were replaced within a Dose condition. This paper does not report any post-hoc corrections for multiple comparisons.

Holze et al. (35) also reported that analyses of data from the same, single experiment were also reported in another paper which focuses primarily on pharmacodynamics and pharmacokinetics and is not assessed in this review. This paper aimed to track subjective effects along with plasma concentrations of LSD. The authors note that the analysis examined peak change from baseline using a repeated-measures ANOVA followed by a Tukey post-hoc test.

Ramaekers et al. (24) reported that they used a general linear model to assess the BSI and CADSS, and that they used mean contrast tests to measure the significance of individual dose effects ANOVA. Additionally, this paper examined the correlations among a set of measures of pain, blood pressure, and dissociation. This paper also does not report effect sizes for contrast analysis. While not described in the *Statistics* section of the paper, some interaction analyses are also included, although those were not statistically significant. Despite running many analyses and not clearly demarcating which are confirmatory and which are exploratory, this paper does not mention performing any post-hoc corrections for multiple comparisons.

Madsen et al. (22) largely focused on pharmacodynamic, pharmacokinetic, and PET scan results. However, this paper also reports taking a linear-regression approach to analyzing the data collected from eight participants. This paper reports descriptive outcome values and Bonferroni-adjusted *p*-values.

de Wit et al. (14) report using a three-way mixed-model ANOVA with drug level as the between-group factor and time as the within-subject factor. In addition, this paper notes that it used peak DEQ scores and that it used maximum change from baseline on “other measures” without further explaining which measures those are. In addition, PANAS scores from days 1–4 were compared using a mixed-model two-way ANOVA, and subjective and behavioral data from session 5 were analyzed using a one-way ANOVA. Finally, DASS scores and total scores were compared using a mixed-model two-way ANOVA across the three treatment groups at screening, before the first drug session and at follow-up. The authors note that for the DEQ, which was not completed pre-drug, only peak scores were used, and on other measures peak change from pre-drug values for each subject were calculated. Notably, this practice favours finding a statistically significant effect. In addition, the paper reports that it was “confirmed that participants in the three groups did not differ at baseline,” although the analyses performed to derive this conclusion remain unknown. Finally, this paper notes that “analyses were not corrected for multiple comparisons,” which presents issues with interpreting its findings (a matter that we further discuss below).

### Appropriateness of inference

This section discusses whether the sum total of the previous sections fits with the way the findings of each paper are interpreted and communicated. We start by describing the importance of cautious and appropriate inference from experimental data, and then review each paper. Again, we start from the papers which, in our opinion, most accurately infer from their data and as in the previous section, we propose improvements at the end of each paragraph rather than at the end of the section.

In the area of psychedelics, inferences are of key importance: a provocative inference is worth a thousand media interviews, and developing a media presence is conducive to obtaining lucrative consulting work. Since the public is hungry for additional information about the utility of psychedelics, scientists stand in the face of much demand for their expertise. The careful and nuanced discussion of research results, including its confounds on generalizability, is an important part of the scientific practice and is discussed below.

Van Elk et al. (15) plausibly connect the finding that most participants broke blind with a state of increased arousal elicited by the microdose. Their discussion also notes that participants had strong expectations for the benefits of microdosing, which are understandable considering that participants were recruited at a psychedelics-enthusiasts convention. The paper dedicates a large section to constraints on generality, including methodological and sample selection issues. However, the paper only dedicates a few sentences to discussing its main findings and does not discuss its null findings (e.g., the finding that psilocybin did not affect body-size perception measures in the context of an awe-inspiring video).

The same team discusses their results more thoroughly in Marschall et al. (17). They propose several explanations for their results and engage with the different results reported in the literature. This paper is also appropriately conservative in drawing strong inferences from its results while also pointing out weaknesses in previous designs, including expectancy effects. However, this paper does not fully address the underlying issue with participants breaking blind in the second block of the experiment: the fact that participants broke blind only in the second block suggests that the amount of psilocybin ingested may have been inconsistent. The authors note that they “had little control over the specific amount of psilocybin that participants consumed, due to natural variability in different batches of psilocybin-containing truffles” (p. 109). The authors also further note this issue in their limitations section, but not to the extent of acknowledging that their results may be unreliable.

Yanakieva et al. (21) go into great detail in attempting to interpret their results, deeply situating their findings in the broader literature. This paper suggests various mechanisms that could be responsible for a change in time perception following the ingestion of microdoses of LSD, including a non-linear dose-dependent response, minimal relevant stimulus intervals, and a deleterious effect on working memory. The authors appropriately weave cautions about interpretation of their results throughout the paper, including the exploratory nature of the work, the small sample size, and that the sample was comprised completely of older adults. They also correctly note that nonsignificant results do not equate to evidence of lack of an effect, but rather that more research is required.

Szigeti et al. (16) reasonably interpret their non-significant results as meaning a lack of effect. However, they misinterpret absence of evidence as evidence of absence: in frequentist statistics, a non-significant effect does not mean that the effect is absent, but rather that the design failed to detect an effect if one is indeed present (68). A non-significant result may occur for various reasons, and the authors mention some of them in their Limitations section. However, the authors ultimately do not qualify their assertion that their “results also suggest that these improvements are not due to the pharmacological action of microdosing, but are rather explained by the placebo effect.” Additionally, while it is possible to ascribe the results to the placebo effect, these findings can be explained using other approaches which may better explain the nuances in the data. One such explanation focuses on the sensitivity of the instruments used. The QIDS, for example, is designed for a depressed population, but the baseline scores were subclinical and only shifted by about 1 point out of a possible 27; the increases in some of the measures of interest were significant but small (e.g., the placebo group improved in life satisfaction by 0.8 ± 1.2 and the microdosing group improved by 1.2 ± 1.2). Perhaps a clinical population would show larger effect sizes. Additionally, the authors note that the quality of the substances participants used was unknown, but they do not consider this issue fundamental to the veracity of their findings: that participants correctly guessed the content of their capsules 72% of the time suggests that the placebo effect is difficult to disentangle from breaking blind in this case, as it is possible that participants dosed inaccurately.

Prochazkova et al. (20) focus on the theoretical contribution of their results. They connect their findings to those of others in the literature to show that their results align with these other extant findings, and they explicitly note that the lack of a control group in their design is an important limitation. They propose that neither learning nor expectancy is likely to be responsible for the effects they found: in this sample, both convergent and divergent measures of microdosing showed improvement post-treatment, but intelligence did not improve. The authors suggest that this may be due to the openness and curiosity-enhancing effect of psychedelics and hypothesize that this activity should be reflected in “high-level prefrontal and associative cortex.” The authors acknowledge the preliminary nature of their results and note that a rigorous randomized double-blind placebo-controlled study is required to validate their findings.

Bershad et al. (18) found a significant effect of the treatment on one subscale of the POMS and on a few 5D-ASC subscales, as well as a significant increase in blood pressure. Despite finding almost exclusively non-significant effects, the authors do not fully discuss most of these findings. Additionally, despite a marginal, non-significant result suggesting that the largest microdose of LSD led to a lower positivity rating of positive images, the authors go to great lengths to explain this effect, thereby treating it as an established (i.e., statistically significant) effect. A similar effect on the number of attempted trials on a measure of creativity is mentioned but not discussed. In the end, this paper does not infer from its results at all and instead presents and situates them with in the literature. Without caveats about the quality of the data presented, this paper suggests that future research should include repeated administration and focus on people who experience negative affect.

Ramaekers et al. (24) take care to couch their results in both the broader psychedelic literature and other pharmacological-pain scholarship without addressing potential weaknesses in their design. The authors suggest their findings are due to psychological rather than neurological causes: small doses of LSD caused lower discomfort ratings and longer exposure to discomfort, likely due to improvements in psychological coping with pain. The authors also suggest that the blood-pressure fluctuations caused by ingesting LSD may have reduced the perception of pain from the experiment. This paper mentions that the differences found would “…survive a conservative Bonferroni tests to correct for multiple comparisons,” but do not perform these tests, and without a clear study plan to demarcate the hypothesis of interest it would be difficult to run this correction. In addition, the paper does not mention other possible issues with its design, such as a training effect over repeated exposures to the uncomfortable stimulus. This paper also does not note the limitation of its relatively small sample size and its lack of heterogeneity (e.g., young age and that all participants had substance-use history).

Hutten et al. (25) appropriately discuss their findings in the context of the broader literature and specifically engage with cases where their findings are confirmed or contradicted. However, this paper does not go into great detail to explain apparent discrepancies between its findings and those of others. This paper also adequately acknowledges its weaknesses, such as a limited number of participants and lack of metabolic, genetic, and other biological tests, noting that more work is required to confirm their findings. They also take care to note a general weakness of the literature inasmuch as different studies often use different measures, making it difficult to compare findings across experiments. This paper also points out an interesting finding: whereas most participants thought they were performing worse on attention tests under the influence of a microdose compared to when in a sober state, they were in fact doing better. It is of note that this Discussion section does not mention the lack of corrections for post-hoc analyses or that the findings should be considered exploratory.

Hutten et al. (25) interpret only some of their findings, focusing on (a) differences between subjective effects in different doses, and (b) their most interesting findings. For example, the paper notes that, interestingly, even though most participants’ PVT performance increased under the influence of a microdose, most participants’ perceived performance decreased. However, the authors do not discuss the fact that the improvements on performance were only in the 5mcg and 20mcg conditions, and that this analysis was conducted post-hoc, following an unexpected interaction that emerged from the data, and should therefore be interpreted with much caution. This paper appropriately notes that more attention modalities is required as microdosing may affect some but not others, and that it is difficult to compare across studies because different measures are often used between studies. In addition, the paper mentions that there may be individual differences in response to the substance consumed, and acknowledges its small sample size. However, the Discussion section does not address the lack of post-hoc corrections for multiple comparisons, which are abound in this paper, meaning that the results reported here should be cautiously interpreted.

de Wit et al. (14) adequately acknowledge that their data showed considerable variability in some of the measures of interest, and that their sample was small. In addition, this paper notes that the tasks used may not be designed to detect the effect of a small dose. However, some of the measures reported in this paper, especially the “Emotional faces task” and “Emotional images task,” are not described, and therefore the way the findings are interpreted cannot be assessed. Finally, since no corrections for multiple comparisons were performed and only peak changes were analysed, this paper clearly aimed to find a maximal effect, which, in addition to the small sample size, means that any effects reported may be spurious.

Family et al. (23) suggest that doses of LSD up to 20 μg were either insufficient to produce any discernible effects in healthy participants on the CANTAB assessment, or that these doses do not have an effect on cognition in a healthy population. Regarding the positive linear relationship found between dose and the dimension of “vigilance reduction” on the 5D-ASC, Family et al. posit that this could be due to the setting of the study: since participants were sitting in beds for 8–12 h, the dimension of “vigilance reduction” may have been impacted. This is a case in which the set and setting were appropriately critically examined, even if only at the stage of interpreting the findings. The authors note that these effects did not impact cognitive performance, which suggests the effects were well-tolerated by participants. However, the Discussion section does not touch upon any of the study’s limitations, including a small heterogeneous sample of older individuals, an assumption that participants were psychedelics-naive, or the exploratory nature of the study. In addition, similarly to Szigeti et al. (16), the authors incorrectly interpret a lack of significant results as suggesting a lack of an effect of microdosing.

## Discussion

This paper aimed to critically assess the current state of the modern literature on microdosing by examining publications from the current wave of research using a few key themes: open science (pre-registration and data sharing), sampling strategies, dose accuracy, design quality, appropriateness of methods; appropriateness of analyses, and appropriateness of the inferences drawn from the data. The methods of some papers –mainly those focused on neuroscience— were not discussed here as the expected standards for neuroimaging data processing and quality control are the subject of an ongoing debate and beyond the scope of the current paper (66, 67). Most papers reviewed here included issues in almost every theme, though some papers showed adherence to rigorous scientific practices across the board. In addition, we reproduced some key analyses from papers when authors made their data available to us. We were able to reproduce some findings, but the shared data failed to reproduce the results reported in a number of papers. Perhaps most disheartening was that several authors refused to share their data, even though sharing data is considered “expected” by many of the journals in which these papers were published.

### Explaining our findings

Our findings suggest that the majority of published work on microdosing is at a preliminary stage, meaning that many of the conclusions should be taken as exploratory. Confidence in research findings should correspond to the quality of the research machinery– including the methods, statistical analyses, and inferences drawn–that produces the results, and as a discipline, we have yet to establish best research practices. This is concerning since in the absence of best practices, or conversely, engagement in “Questionable Research Practices,” (QRPs) (69), become rife. In the context of microdosing research, the current state of the literature is that the knowledge accumulated so far should generally be considered preliminary and exploratory. We are particularly concerned about the lack of pre-registration coupled with biased sampling strategies, designs with unknown doses, designs that used *ad-hoc* measures and interventions, analyses that do not follow the best statistical practices, and inferences drawn from data that are uninformed by the philosophy of science, all of which produce a body of knowledge that hinders our ability to understand the putative effects of microdosing psychedelics.

Good science includes planning one’s work, making concrete hypotheses, and collaborating with one’s peers by sharing data, as the scientific enterprise is a collaborative one. To produce good science, one should also consider how their work contributes to the literature at large. However, in the current publishing climate, there are arguably few incentives to work rigorously, collaboratively, and thoughtfully. Out of the 15 papers reviewed here, only two pre-registered their designs and hypotheses, and both were part of the same project. In the absence of an external incentive structure and education about its importance, scientists may consider the process of pre-registration unnecessarily onerous and restrictive despite its well-documented benefits (70). This issue has already been called to the attention of microdosing researchers and a research checklist has been provided (3).

We found an alarming trend in terms of data sharing: out of 15 papers reviewed, the data for four were available online and, of the remaining 11 papers, only two shared their data. Since publishing in its current form is a relatively solitary pursuit, there are no inherent incentives for scientists to share their data, and recent findings suggest that researchers often do not share their data even when journal policies require them to do so (71), as was the case with some of the data requests made for this paper. Notably, the lack of pre-registration and collaboration is useful for the publication industry: without planning, surprising–but erroneous–results are more likely to be published, and without collaboration, the quantity of publications increases. It may be that young disciplines in which scientists are attracted to a publishing “gold rush,” as is the case with the microdosing discipline, are particularly vulnerable to these practices. We therefore recommend following the work of Marschall et al. (17) as a blueprint for data sharing, and the corresponding author of this paper extends an open invitation to discussing the principles of Open Science with those who are interested.

The microdosing research reviewed here also generally tended to lack adequate sampling strategies. Most studies reviewed either recruited very small samples of individuals who do not represent the general population, were unblinded, or were otherwise biased. In general, the papers reviewed here did not report *a priori* power analyses and thus, in some studies, small but real effects may not have been detected due to low power. Meanwhile, other studies with statistically significant effects may have reported an exaggerated effect size because only an exaggerated effect could be detected with such low power. These confounds make inferring from the results in this literature untenable. This is particularly noteworthy as a dose–response curve for microdosing has yet to be identified. Indeed, the placebo effect may play an outsized role in the case of psychedelics (10, 37), making the importance of controlling for expectancy particularly important (39). If journals and reviewers were to require sampling strategies and power analyses, the quality of the data obtained would be substantially higher.

Most (but not all) studies, especially those using psilocybin, did not accurately dose their participants. It is understandable that while dosage accuracy is normally a tenet of pharmacological research, ethnographical and quasi-experimental research cannot control for this factor. Additionally, since mushrooms vary in the amount of psilocybin they contain, it is difficult to accurately and consistently identify the amount of psilocybin in a study. It is therefore important to add caveats and interpret results accordingly under these circumstances. If the dosage is inaccurate or inconsistent, the aforementioned concern for undetected true effects or exaggerated significant effects should be noted in publications, with adequately conservative interpretations following from the findings. While most studies reviewed here did not note any such caveats, some were mindful of the limitations that inaccurate dosage pose. The overall picture, however, suggests that LSD studies dosed more accurately and that the results from psilocybin studies should be taken with a grain of salt.

The designs of the majority of papers reviewed here were likely inadequate to systematically and accurately examine the effects of their microdosing interventions. We assessed whether studies were placebo-controlled and whether set and setting were addressed. While we appreciate that running a trial using a scheduled substance such as psilocybin or LSD is no small feat, and while the earliest studies used samples of convenience in uncontrolled settings, we note that most microdosing papers reviewed here did not use a placebo-controlled design (6). Without a placebo-controlled design, however, all inferences should be drawn carefully, cautiously, and with appropriate caveats. Additionally, the set and setting in which psychedelics are used are frequently predictive of their impact (72), which may be of particular note in the case of microdosing (10). Unfortunately, though, most papers reviewed here did not report any information regarding their participants’ set and setting.

When studying a new area of research where effects are unknown, it is particularly important to use validated measures so that results may be trusted; some of the papers reviewed here failed to engage this practice. The maxim we suggest is “when studying unusual phenomena, use well-validated methods,” and this is of special import when the phenomena are hotly contested and vulnerable to biased reporting. Since various stakeholders are arguably interested in provocative results, using well-validated methods to measure the constructs of interest is imperative for producing results that are trustworthy. This was not the case in several of the papers reviewed here, which used *ad-hoc* measures, measures intended for large-doses, or even measures that were developed to index entirely different constructs than the constructs that were purportedly measured. As a result, many of the findings in the microdosing literature are uninterpretable because measure quality is unknown or unacceptable. We hope that in the future specific tools will be developed for the study of microdosing and will be able to capture the construct of interest.

The quality of the analyses performed on the data examined here was disheartening in a number of cases. As described above, many of the statistical methods applied in the reviewed papers were inadequate, resulting in a wide gap between failing to detect real effects and detecting exaggerated effects, rendering findings inconclusive. Inappropriate statistical methods were common, including repeated analyses of the same data published in different papers, a lack of correction for multiple comparisons, a lack of reporting regarding excluded participants, and the use of confirmatory strategies for exploratory analyses. As such, the bulk of the analyses reported in the literature should be considered exploratory. Existing findings may serve as tentative hypotheses to consider in future research, but should not be considered as supporting any particular hypothesis.

Inferences drawn from the data were often problematic as well, but were generally acknowledged as preliminary. Although many participants broke blind in the studies that collected these data, the inferences drawn still largely focused on the substance itself, with little (if any) regard for set and setting, despite the importance of these constructs (10, 37, 73). Reporting of the outcomes of analyses is frequently motivated such that some “almost significant” results are reported and discussed at length, while other non-significant results are not discussed at all, or are even treated as confirming the non-existence of an effect. There was little discussion of the relevance of participants’ prior psychedelic use, and little discussion of other factors that may have affected the results. When making inferences from data, researchers should consider the entire causal chain from study planning, Open Science policy, sampling strategy, dose accuracy, design, measures, and analysis; yet, with one exception (17), none of the papers considered these limitations in their discussions.

Of final note, in the context of microdosing, the need for collegial collaboration is seemingly quite pressing. This paper would not be needed if the culture in the field included communicating with each other in terms of research plans, asking for advice, and following best practices. Thus, moving forward, we propose the establishment of a consortium of psychedelic research, which could be a hub for discussing experimental design, ethical conduct, and open science. Scientists who join the consortium would gain access to their peers’ input on their work, helping improve it before data collection commences, so that every study would be as informative as possible. An additional benefit would be the inclusion of simple, standardized measures for specific indications, such that data from different studies on the same indication can be pooled and analyzed together. Perhaps most importantly, members of the consortium would pledge to practice Open Science, including pre-registration, open data (as much as possible), and constraints on generality. By creating an *agora* where scientists can cross-pollinate and keep each other accountable, we may be able to turn the tide of psychedelic research from quantity to quality.

## Conclusion

At the conclusion of this review, we are concerned that psychedelics may be in the midst of a great popularization, but that they risk being poorly understood and misapplied, creating a “McPsychedelics” akin to “McMindfulness” (74). The popularity of mindfulness provides a cautionary tale: while mindfulness has become a household word, the literature is filled with repeated alarm-raising about poor research quality, poor control conditions, and overlooked risks (11, 75–77). Mindfulness enjoys a very popular house of cards; we hope for a brighter future for psychedelics research, and contend that the research norms we establish now will have an outsized impact on future research. As psychedelics become more medicalized and legalization efforts continue, the risk for repeating the past increases (78). Furthermore, considering the legal landscape of the 20th century in which psychedelics quickly shifted from potential wonder-drugs to criminalized substnaces, we believe that a sound scientific foundation will help inform policymakers in the event that substance schedules are re-evaluated. It is our responsibility to do the best science we can rather than the fastest science that can get past peer review.

## Data availability statement

The original contributions presented in the study are included in the article/supplementary material, further inquiries can be directed to the corresponding author.

## Author contributions

RP collated, wrote, and edited the manuscript. TA consulted on the writing and provided edits. EF conducted the preliminary literature review and provided edits. YA communicated with researchers in the field, conducted the analysis, and provided edits. MH helped form the idea, provided critical comments throughout, and helped with edits. MG provided comments throughout, especially to do with neuroscience, and provided edits. PS provided comprehensive edits and mentoring throughout. All authors contributed to the article and approved the submitted version.

## Footnotes

^1^YA has no background in psychedelic research but does have a strong background in quantitative analyses and was not debriefed about the studies prior to performing any analyses.
